# Comparison of Dental Anxiety While Visiting Dental Clinics before and after Getting Vaccinated in Midst of COVID-19 Pandemic

**DOI:** 10.3390/vaccines10010115

**Published:** 2022-01-13

**Authors:** Abhishek Lal, Sara Saeed, Naseer Ahmed, Mohammad Khursheed Alam, Afsheen Maqsood, Mahmud Uz Zaman, Huda Abutayyem

**Affiliations:** 1 Department of Prosthodontics, Altamash Institute of Dental Medicine, Karachi 75500, Pakistan; abhishekdarshan@yahoo.com (A.L.); dr.sara.s.khalid@gmail.com (S.S.); 2 Prosthodontics Unit, School of Dental Sciences, Health Campus, Universiti Sains Malaysia, Kota Bharu 16150, Kelantan, Malaysia; 3 Department of Preventive Dentistry, College of Dentistry, Jouf University, Sakaka 72345, Saudi Arabia; 4 Center for Transdisciplinary Research (CFTR), Saveetha Dental College, Saveetha Institute of Medical and Technical Sciences, Saveetha University, Chennai 600077, India; 5 Department of Public Health, Faculty of Allied Health Sciences, Daffodil International University, Dhaka 1207, Bangladesh; 6 Department of Oral Pathology, Bahria University Dental College, Karachi 75530, Pakistan; afsheenmaqsood.bumdc@bahria.edu.pk; 7 Oral and Maxillofacial Surgery and Diagnostic Sciences Department, College of Dentistry, Prince Sattam Bin Abdulaziz University, Al-Kharj 16245, Saudi Arabia; m.zaman@psau.edu.sa; 8 Department of Orthodontics, College of Dentistry, Ajman University, Ajman 346, United Arab Emirates; h.abutayyem@ajman.ac.ae

**Keywords:** coronavirus, COVID-19 pandemic, dental clinics, dental anxiety, vaccines

## Abstract

Vaccination is critical to control the rate of coronavirus transmission and infectivity. Dental practices are a high-risk area for contracting coronavirus; this fact generates psychological disturbances amongst patients. In this study, we aimed to assess the levels of anxiety of patients while visiting dental practices before and after getting vaccinated. This cross-sectional study was carried out between March and December 2021. An electronic survey was distributed among the vaccinated individuals who visited dental clinics before and after getting vaccinated. The survey consisted of the following four parts: demographic characteristics, questions related to coronavirus, and anxiety scores before and after getting vaccinated. SPSS-25 was used to perform the statistical analysis, where paired *t*-test was used to compare the anxiety scores, and Mann–Whitney U test to assess the association of gender with anxiety scores. A *p*-value of ≤0.05 was considered to be statistically significant. A total of 400 vaccinated individuals participated in this study, with a response rate of 88.23%. The majority of the respondents (71.0%) did not test positive for coronavirus. More than half of the participants (54.0%) reported to not be suffering from any coronavirus-related symptoms. About 100 (25.0%) of the individuals stated that dental clinics are an environment in which there is a high risk of contracting coronavirus. In regards to the comparison of the mean MDAS scores of the participants before and after getting vaccinated, a significant difference (*p* = 0.001) was found. Vaccination has been recommended for all eligible individuals to control the transmission and infectivity of coronavirus. Vaccinations have decreased the dental anxiety of patients while visiting dental clinics. However, the protective measures are still valid and should be followed, regardless of the vaccination status.

## 1. Introduction

December 2019 marked the beginning of the currently known coronavirus pandemic in Wuhan, China, which is currently on the rise and is a major public health challenge. Various healthcare organizations, such as World Health Organization (WHO) and the Centre for Disease Control and Prevention (CDC), have been working since the beginning to provide information regarding precautionary measures and disseminate information that is needed to know more about coronavirus. SARS-CoV-2 is a rapidly spreading virus that primarily transmits through respiratory droplets that can be inhaled from an infected person by a healthy person, by coughing and sneezing [1]. Since, at the present moment, different variants of coronavirus are present globally, the incubation period, along with the pathophysiology of the variants, varies to some extent [2]. Nonetheless, the symptoms of coronavirus-infected patients include fever, dry cough, sore throat, loss of smell and taste sensations, myalgia, and stomach upset [3]. However, those with underlying systemic co-morbidities and the elderly are more prone to developing a severe infection if they contract the virus [4]. To combat this global public health challenge, many organizations have developed vaccines against coronavirus that work based on a different mode of action, but the primary goal is to provide a strong immune response to reduce the chances of developing a severe infection if a person does contract the virus [5]. The first person to receive the coronavirus vaccine was a 91-year-old female in the United Kingdom [6]. After that, different organizations formulated coronavirus vaccines that were administered throughout the world, starting with the elderly and those with underlying co-morbidities. Later on, the eligibility criteria for the vaccine were expanded, and, currently, those above 18 years of age can get themselves vaccinated. Currently, in Pakistan, an average of 502 cases of coronavirus are reported each day, with 1,287,235 infections and 28,943 deaths reported since the beginning of the pandemic [7]. In terms of vaccinations in Pakistan, about 159,058,469 doses of vaccines have been administered so far [7]. Since the primary mode of transmission for coronavirus is through respiratory droplets, dental practices are at the frontline in terms of potentially contracting the virus, since most dental procedures generate aerosols [8]. This has led to WHO- and CDC-approved guidelines for the appropriate use of personal protective equipment (PPE) for frontline healthcare workers, including dental surgeons. Similarly, WHO has also issued guidelines for the general population for the use of face masks and hand sanitization to reduce the chances of contracting the virus. Since a dental practice is an environment in which there are greater chances of contracting the virus, this has led to increased anxiety amongst patients when visiting or planning to visit the dentist [9]. Before the vaccination program had commenced, the general population primarily avoided visiting the dentists because of the anxiety associated with contracting coronavirus [10]. Only in the case of emergencies was a visit to the dentist inevitable for the patients [11]. Due to the COVID-19 pandemic, the dental practice was considered an environment of high occupational hazard because of aerosols and oral fluids, additionally contributing to the transmission of the virus [12]. Furthermore, since the rate of transmission and infectivity of coronavirus is known to be high, this further aggravates the anxieties of the dentists, as they are afraid to transmit coronavirus to their friends and family if they are COVID-19 positive. Keeping that in mind, vaccinations are considered the most beneficial tool against coronavirus, to limit its spread. Previously, vaccinations have been known to control the transmission of various viruses, such as measles, mumps, and rubella (MMR) [13]. Therefore, in order to manage the anxieties associated with contracting coronavirus, for the patients as well as the dentists, vaccinations against it have been recommended by renowned organizations to combat the morbidities and mortalities associated with coronavirus. In this study, we aimed to evaluate the anxiety levels of patients before and after getting vaccinated whilst visiting the dental practice amid the COVID-19 pandemic. 

## 2. Materials and Methods

### 2.1. Study Design and Sample Size

In this cross-sectional study, adults who resided in Pakistan were invited to participate. The data from the participants were collected after ethical approval. The study was conducted by the Declaration of Helsinki and approved by the Ethical Review Committee of Altamash Institute of Dental Medicine, Karachi, Pakistan (AIDM/ERC/12/2021/01). The participants in this study were recruited using a convenience sampling method. Participants who showed a willingness to participate in this study were asked to give verbal and written consent. The objective of this study was carefully explained to the participants and their queries about the research were answered. An online well-structured questionnaire was formulated using Google forms and distributed to the participants using social media platforms, such as Facebook^©^, Snapchat^©^, WhatsApp^©^, Twitter^©^, and also through emails. Keeping in mind the current coronavirus pandemic situation, hard copies of the questionnaire were not sent to the participants to avoid cross-infection. The sample size of this study was calculated using Open-epi software. Keeping the confidence interval at 95% and desired percentile at 5%, the estimated sample size calculated was 400 (*n* = [Z12-α/2.p.q]/d2) [14].

### 2.2. Questionnaire Design and Distribution

In this study, the questionnaire consisted of five parts. In the first part, demographic characteristics of each participant, such as age, gender, education, and occupation, were recorded. The second section consisted of questions related to the coronavirus infection, such as the history of contracting coronavirus, history of experiencing coronavirus symptoms, methods of precautionary measures used for protection against coronavirus, and being vaccinated or not against coronavirus. Furthermore, in the third part, questions regarding dental history, i.e., history of dental pain, reason of concern while visiting the dental clinic, and willingness to visit a dentist in case of a dental emergency, regardless of being vaccinated or not. Next, the dental anxiety of the participants was recorded before getting vaccinated while visiting a dental clinic. Lastly, the same patients were followed up to evaluate and record their dental anxiety levels before and after getting vaccinated while visiting a dental clinic. All the questions in the questionnaire were kept in English and their translation in Urdu.

### 2.3. Anxiety Measurement

**Figure 1:**
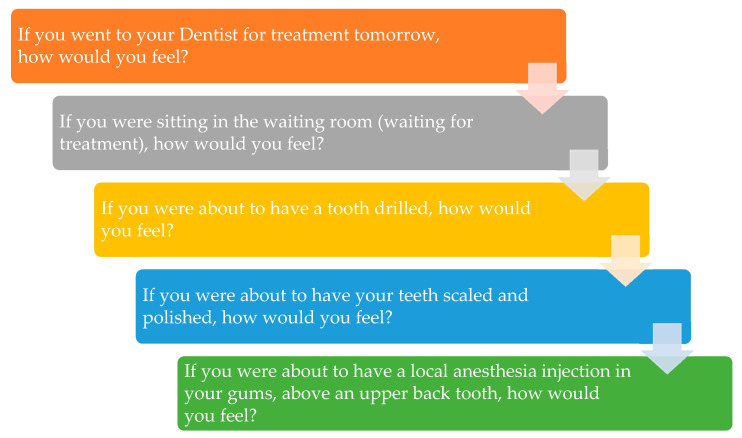
Modified dental anxiety scale.

Modified dental anxiety scale (MDAS) is a commonly used scale to measure the dental anxiety of patients. The studies have reported internal consistency of α = 0.94 [15]. The modified dental anxiety scale measures dental anxiety using five questions, with each question having five options in Likert scale rating, which are 1 = not anxious, 2 = slightly anxious, 3 = fairly anxious, 4 = very anxious, and 5 = extremely anxious, as presented in Figure 1. Each option has its numerical value, which, when added, represents a total score for each participant. The total score ranges from 5 to 25, and a cut-off value of 19 is kept, which suggests high clinically significant dental anxiety and possible dental phobia. For each participant, MDAS scores before and after getting vaccinated were calculated and compared.

### 2.4. Inclusion and Exclusion Criteria

Participation in this study was based on assessing the dental anxiety of the adults while visiting dental clinics before and after getting vaccinated. The adult patients, being above 18 years of age, who visited the dental clinics were included in this study. Children and those who were not vaccinated were excluded from this study. The information collected regarding participants was kept anonymous and confidential throughout this study.

### 2.5. Statistical Analysis

The data analysis was carried out through SPSS Statistical software version 25. Descriptive statistics were performed to calculate the mean, frequency, percentage, and standard deviation of demographic characteristics. Mann–Whitney U test was used to assess the effect of gender on the anxiety levels of the patients. Paired *t*-test was used to compare the MDAS scores before and after vaccination, along with a comparison to the demographic characteristics. The Spearman’s correlation test was used to assess the relation of age, education, and occupation with anxiety scores before and after vaccination. A *p*-value of ≤0.05 was considered significant.

## 3. Results

In this study, out of the 400 participants, 159 (39.7%) were males and 241 (60.3%) were females. The majority of the 300 (75.0%) participants belonged to the age bracket of 18–30 years, and 56 (14%) were 31–40 years old. Regarding education status, most of the participants (46.0%) were undergraduates, while 25% had a graduate-level education, with students and healthcare workers being the most common occupation amongst the participants, as presented in Table 1.

Regarding medical history, most of the 280 (70.0%) participants did not suffer from any medical condition. Regarding being tested positive for coronavirus, the majority of the 284 (71.0%) respondents did not test positive for coronavirus. Just over half of the participants (216; 54.0%) reported to not be suffering from any coronavirus-related symptoms. For protection against contracting coronavirus, the majority of the participants (300; 75.0%) performed all the necessary preventive measures that have been recommended by well-renowned healthcare organizations.

Regarding being afraid of contracting coronavirus, the majority of the 288 (72.0%) participants reported having the fear of contracting the virus before getting vaccinated. However, after getting vaccinated, about 232 (58.0%) participants reported having the fear of contracting coronavirus. Regarding the concern, while visiting dental clinics, about 100 (25.0%) of the individuals stated that dental clinics are an environment in which there is a high risk of contracting coronavirus.

**Figure 2:**
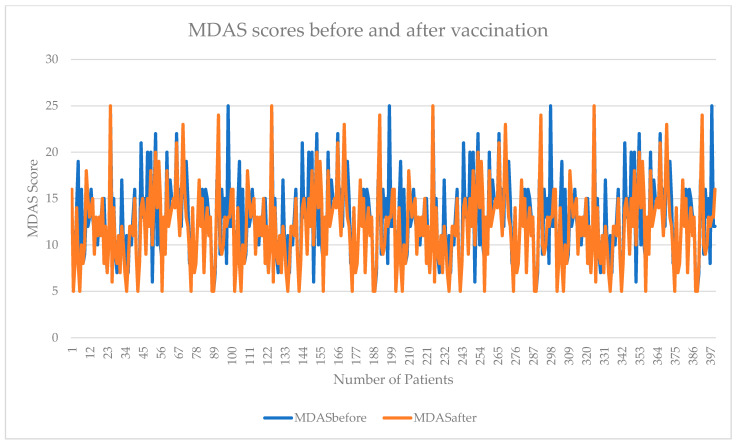
Comparison of anxiety scores of patients before and after vaccinations. MDAS: Modified dental anxiety scale.

Regarding the dental anxiety of the participants, while visiting the dental clinics before and after getting vaccinated, out of 400 participants, 40 (10.0%) of the individuals suffered from severe dental anxiety before they got themselves vaccinated. After getting vaccinated, when the participants visited the dental clinics, about seven (1.75%) participants reported suffering from dental anxiety. The differences in the mean MDAS scores of the participants before and after getting vaccinated, while visiting the dental clinics, are presented in Figure 2.

Regarding the comparison of the mean MDAS scores of the participants before and after getting vaccinated, using paired *t*-test, a significant difference was noted (*p* = 0.001), as shown in Table 2.

A significant relationship (*p* = 0.001) was found between gender and anxiety levels. The females were more concerned about contracting coronavirus, as presented in Table 3.

Regarding the comparison of age, education, and occupation with the before and after vaccination anxiety scores, age, and occupation had a significant relationship (*p* < 0.05) with anxiety. However, no significant association (*p* = 0.066) was found between education and anxiety levels after vaccination in participants as shown in Table 4.

## 4. Discussion

Dental anxiety is a common finding amongst patients visiting dental clinics. This fear of dental clinics is further exacerbated by the current pandemic situation, as dental clinics are an environment in which there is a high risk of contracting the virus [16]. Most dental procedures generate aerosols, such as dental extractions, root canal treatments, ultrasonic scaling, and fillings. These aerosols then place the entire dental practice at risk of contracting the virus if proper preventive measures are not adhered to.

In our study, where we included only coronavirus-vaccinated individuals, most of the individuals did not test positive for coronavirus, but just less than half of the participants did experience coronavirus-related symptoms. Since many of the symptoms of coronavirus appear to be similar to the common cold, many individuals did not undergo PCR tests for coronavirus. However, many cases of coronavirus have been reported where the individuals reported no such symptoms [17]. Most of the participants in our study were afraid of contracting the virus since the rate of transmission and infectivity of coronavirus is high. Similar results have been reported in a study where individuals did suffer from the distress of contracting coronavirus [18].

Vaccinations have been manufactured by different pharmaceutical companies around the globe, with different vaccinations having a different mechanism of action to provide immunity to the individuals receiving their shots. To control the rate of transmission and infectivity of coronavirus, vaccination has been recommended for all eligible individuals. Healthcare professionals, such as dental surgeons, are among the frontline workers that are at high risk of possibly contracting coronavirus [19]. Therefore, it has been made mandatory for dentists to get themselves vaccinated. Similarly, eligible patients are also recommended to get themselves vaccinated, especially if they visit the dental practice, as their risk of contracting coronavirus, as well their mortality rates, are high as compared to vaccinated individuals [20].

Since the dental practice is an environment in which there is a high risk of contracting coronavirus, there has been hesitation among patients as to whether they should visit the dental practice or not. About 40% of the patients in our study considered the dental clinic as a high-risk environment for contracting coronavirus, along with having the fear of contracting the virus itself. Such findings correspond with a study by Ibrahim et al., where the patients also experienced fear of contracting the virus while seeking dental care [21]. Furthermore, the majority of the patients in our study reported that they would consider visiting the dental practice in cases of emergency, regardless of being vaccinated or not. These results have also been concluded in a study in the literature, where most of the patients preferred visiting dental clinics in cases of emergency [22].

An array of information is available to individuals about the transmission, morbidities, mortalities, and ways of preventing coronavirus. In this study, the patients generally avoided the dental practice, due to the patients’ adapted avoidance behavior towards the dental practices, unless an emergency arises that necessitates a visit to the dentist [23]. In our study, the patients generally reported suffering from high anxiety before they got vaccinated against coronavirus and visited the dental practice. These results correspond with a study by Olmo et al., where the patients reportedly experienced higher levels of anxiety while visiting the dental practice [24].

Since vaccinations are now available, it has been made mandatory for healthcare professionals, such as dentists, to get themselves vaccinated against coronavirus, as they are directly exposed to aerosols. Similarly, eligible patients are also recommended to get themselves vaccinated to control the transmission of the virus. In our study, after the patients got themselves vaccinated, a decrease in the levels of anxiety was noted amongst the patients who previously experienced higher levels of anxiety before getting vaccinated. Such findings have also been concluded in a study by Chen et al., where a significant decrease in the anxiety and depression of the patients was found after they got vaccinated against coronavirus [25]. Although the vaccine does offer some psychological comfort for the individuals, it does not completely prevent re-infection with coronavirus [26,27]. Therefore, despite the vaccination status, it has been recommended to follow the protective measures against coronavirus.

Regarding gender, in our study, females experienced higher dental anxiety when they were visiting the dental practice amid the coronavirus pandemic, as compared to males. Such findings correspond with studies in the literature that conclude that females tend to suffer more from dental anxiety as compared to males [28,29]. However, this is not always the case, as a study reported that no significant association exists between gender and dental anxiety levels [30].

Various symptoms are experienced by patients suffering from coronavirus, and many individuals report being asymptomatic, with a global prevalence of such asymptomatic cases of 0.25% amongst the tested population and 40.50% amongst the confirmed COVID-19 cases. The reported pooled percentage of asymptomatic infections was higher in North America (46.32%), followed by Europe (44.18%), and less in Asia (27.58%) [31,32,33]. Despite the strengths of this study, such as including a good sample size of patients and follow-up of the patients before and after getting vaccinated while visiting the dental practice, this study was met with some limitations. Firstly, some patients were not afraid of contracting the virus, despite their vaccination status, so this can affect the anxiety scores of the individuals. Lastly, since a convenience sampling method was used, there is some risk of biasness.

## 5. Conclusions

The coronavirus pandemic has been on the rise, with different mutations being found globally. To tackle it, vaccinations have been recommended to individuals to decrease the anxieties associated with contracting the virus. After administration of the vaccine, the anxiety levels of the patients did result in a significant reduction when visiting the dental practices, since the vaccines decreased the chances of developing severe coronavirus infection. However, preventive measures should still be implemented by the patients, as well as the dental surgeons in the dental practice, as the vaccines do not co-eliminate the chances of contracting the virus.

## Figures and Tables

**Table 1:** Distribution of demographic details of participants (*n* = 400).

Demographics	Number/Percentage
Age:	
18–30 years	300 (75.0)
31–40 years	56 (14.0)
41–50 years	28 (7.0)
51–60 years	8 (2.0)
Above 60 years	8 (2.0)
Gender:	
Male	159 (39.7)
Female	241 (60.3)
Level of Education:	
Undergraduate	170 (42.5)
Graduate	120 (30.0)
Post-graduate	74 (18.5)
Below Undergraduate	36 (9.0)
Occupation:	
Student	120 (30.0)
Healthcare Professional	118 (29.5)
Engineer	10 (4.0)
Business	42 (9.0)
Teacher	30 (10.5)
Unemployed	48 (12.0)
Others	32 (8.0)

**Table 2:** Comparison of mean MDAS scores before and after getting vaccinated.

	Mean	*N*	Std. Deviation	Std. Error Mean	t	df	*p*-Value
Before getting vaccinated MDAS Score	12.73	400	4.524	0.226	4.97	399	0.001
After getting vaccinated MDAS Score	11.99	400	4.211	0.210			

t: measures the size of the difference relative to the variation in the sample data, df: difference, *N*: number of participants.

**Table 3:** Relationship of anxiety scores in both sexes *(n* = 400).

	Gender	*N*	Mean Rank	Z	*p*-Value
Before getting vaccinated MDAS Score	Males	159	176.88	−3.329	0.001
	Females	241	216.09		
	Total	400			
After getting vaccinated MDAS Score	Males	159	176.81	−3.340	
	Females	241	216.13		
	Total	400			

MDAS: Modified dental anxiety score, Z: difference of the sum of mean ranks between the groups, *N*: number of participants.

**Table 4:** Comparison of age, education, and occupation with anxiety scores.

Variables		Before Vaccination MDAS Score	After Vaccination MDAS Score
Age	Correlation Coefficient	0.172	0.198
	*p*-value	0.001 *	0.001 *
Education	Correlation Coefficient	0.150	0.092
	*p*-value	0.003 *	0.066 **
Occupation	Correlation Coefficient	0.205	0.197
	*p*-value	0.001 *	0.001 *

MDAS: Modified dental anxiety score, * *p* < 0.05 a significant difference, ** *p*-value > 0.05 no significant difference.

## Data Availability

The data presented in this study are available on request from the corresponding author.
